# Supplementary material for: Virus-like Particle Vaccine for Feline Panleukopenia: Immunogenicity and Protective Efficacy in Cats
Source: Vaccines (Basel). 2025 Jun 25;13(7):684. doi: 10.3390/vaccines13070684 (PMC12299992; doi:10.3390/vaccines13070684)
Supplement: Supplementary file 1 [file vaccines-13-00684-s001.zip › vaccines-3675027-supplementary.pdf]

Table S1. The Immunogenicity induced by FPV VLPs Vaccine at 21 days post-immunization

Group	Group1 (VLPs 45μg)	Group2(VLPs 15μg)	Group3 (VLPs 5μg)	Group4 (PBS)
HI antibody titer	11log$_2$,10log$_2$,11log$_2$,12log$_2$,11log$_2$	10log$_2$,11log$_2$,10log$_2$,11log$_2$,10log$_2$	9log$_2$,8log$_2$,9log$_2$,9log$_2$,8log$_2$	0,1log$_2$,0,0,0
neutralizing antibody titers	10log$_2$, 9log$_2$, 9log$_2$,8log$_2$,10log$_2$,	9log$_2$,10log$_2$, 8log$_2$,9log$_2$, 10log$_2$,	6log$_2$, 5log$_2$, 6log$_2$,4log$_2$, 6log$_2$,	0,1log$_2$,0,0,0

Table S2: Clinical Symptoms During Challenge in VLP Group 2and Control Group 4

Group	Clinical Symptoms	Day0	Day1	Day2	Day3	Day4	Day5	Day6	Day7	Day8	Day9	Day10
Group2 (VLPs 15μg) 6#-10#	Loss of appetite	-	-	-	-	-	-	-	-	-	-	-
	Depression	-	-	-	-	-	-	-	-	-	-	-
	Vomiting	-	-	-	-	-	-	-	-	-	-	-
	Diarrhea	-	-	-	-	-	-	-	-	-	-	-
	Fever	-	-	-	-	-	-	-	-	-	-	-
Group4 (PBS) 16#-20#	Loss of appetite	-	-	-	-	16#-20#	16#-20#	16#-20#	16#-18#，20#	16#，18#，20#	16，18#，20#	16#，18#
	Depression	-	-	-	-	-	19#,20#	17#,19#,20#	16#,20#	16#,18#,20#	16#,18#	16#,18#
	Vomiting	-	-	-	-	-	-	17#	-	20#	-	-
	Diarrhea	-	-	-	-	-	17#	19#	16#，20#	16#,18#	-	-
	Fever	-	-	-	-	-	19#	-	16#	-	18#	-

Note: 19# cats died on the 6th day post-challenge;17# cats died on the 7th day post-challenge;20# cats died on the 9th day post-challenge.

– indicates that no abnormalities were observed in this group on that day. A cat's body temperature exceeding 39.5°C is considered a fever.
